# Volumetric burden of metastatic lesions drives outcomes in patients with extracranial oligometastatic disease

**DOI:** 10.1002/cam4.4332

**Published:** 2021-10-20

**Authors:** Yilin Cao, Hanbo Chen, Arjun Sahgal, Darby Erler, Serena Badellino, Tithi Biswas, Roi Dagan, Matthew C. Foote, Alexander V. Louie, Ian Poon, Umberto Ricardi, Kristin J. Redmond

**Affiliations:** ^1^ Department of Radiation Oncology and Molecular Radiation Sciences Johns Hopkins University School of Medicine Baltimore Maryland USA; ^2^ Department of Radiation Oncology Sunnybrook Health Sciences Centre University of Toronto Toronto Ontario Canada; ^3^ Department of Oncology University of Turin Turin Italy; ^4^ Department of Radiation Oncology University Hospitals Seidman Cancer Center Cleveland Ohio USA; ^5^ Department of Radiation Oncology University of Florida College of Medicine Gainesville Florida USA; ^6^ Department of Radiation Oncology Princess Alexandra Hospital University of Queensland Brisbane Queensland Australia

**Keywords:** metastasis‐directed therapy, oligometastatic disease, oligometastatic outcomes, SBRT, volume of metastases

## Abstract

**Background:**

We hypothesized that the total volume of metastases at initial oligometastatic (OM) presentation to stereotactic body radiation therapy (SBRT) is an important prognostic factor that can refine the definition of OM disease.

**Methods:**

Patients with extracranial oligometastatic cancer (≤5 lesions) treated with SBRT were included in an international multi‐institutional database. Multivariable Cox and competing risks regression models were used to determine the relationship between distant progression‐free survival (DPFS), widespread progression (WSP), and overall survival (OS) with the total planning target volume (PTV) at initial OM presentation to SBRT. All models were adjusted for histology, pre‐SBRT systemic therapy, osseous‐only lesions, and number of metastases.

**Results:**

In total, 961 patients were included. The median follow‐up was 24.4 months (IQR: 13.8–37.5). Total PTV had a significant effect on DPFS in the first 18 months after SBRT and was most profound in the first 6 months, when each twofold increase in total PTV conferred a 40.6% increased risk of distant progression (*p *< 0.001). Each twofold increase in total PTV increased the risk of WSP by 45.4% in the first 6 months (*p *< 0.001). Total PTV had a significant effect on OS in the first 2 years after SBRT, with each twofold PTV change increasing the risk of death by 60.7% during the first 6 months (*p *< 0.001) and by 34% thereafter (*p *< 0.001). Exploratory gross tumor volume (GTV) analysis confirmed the PTV‐based observations.

**Conclusion:**

The total volumetric burden of metastases at initial OM presentation to SBRT is strongly and independently prognostic for the risk of distant and widespread progression and survival. We propose that this metric should drive the definition of OM disease and guide treatment decision‐making.

## Graphical abstract

This large international, multi‐institutional series of SBRT to extracranial oligometastases demonstrates total volume of metastatic lesions as a strong, independent prognostic factor for cancer outcomes. This metric can redefine oligometastatic disease beyond number of lesions and refine patient selection for an increasingly important treatment paradigm.

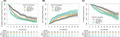

## 1 INTRODUCTION

There has been increasing interest in the oligometastatic (OM) state since the concept's initial introduction by Hellman and Weischelbaum.^1^ The concept is that the presence of metastasis is not purely a binary state but rather a spectrum between localized primary tumors and widespread metastatic disease which influences cancer outcomes and treatability. The hope is that within this spectrum, the opportunity for long‐term control or even cure may exist if local control can be achieved to limited sites of metastatic disease. Randomized phase II trials across several primary tumor histologies have demonstrated the promise of this concept, reporting benefit to endpoints such as progression‐free survival (PFS) and overall survival (OS) when employing metastasis‐directed radiation therapy (MDRT).^2^, ^3^, ^4^ Stereotactic body radiation therapy (SBRT) has been increasingly utilized as the radiation therapy modality of choice in these situations. A survey of >1000 international radiation oncologists published in 2017 reported that 84% of responders who use SBRT to treat extracranial OM disease cited perceived treatment response and durability as the primary reason for this practice paradigm.^5^

In 2020, ESTRO (European Society for Radiotherapy and Oncology) and ASTRO (American Society for Radiation Oncology) published a collaborative consensus guideline for identifying and treating OM disease with radiation therapy.^6^ They reported that the definition of OM disease is typically based on the number of imaging‐detected metastases, at a consensus maximum of five lesions. However, they acknowledged that significant limitations exist in this definition, including inconsistency in the specific number of lesions in the existing literature (most typically 3–5 or fewer) and the absence of additional clinical or molecular biomarkers to aid in the classification.

We previously reported outcomes of SBRT in 1033 oligometastatic patients compiled from 6 academic high‐volume international centers.^7^ Here, we report a sub‐analysis to investigate the hypothesis that the total volumetric burden of metastases at initial OM presentation to SBRT is an independent prognostic factor for oncologic outcomes such as distant progression‐free survival (DPFS), widespread progression (WSP, i.e., >5 new lesions), and overall survival.

## 2 METHODS

### 2.1 Patient selection for master database

Six radiation oncology departments across four countries created a pooled database of patients treated with SBRT to extracranial oligometastatic lesions between January 2008 and December 2016. Patients meeting the following criteria were included: (1) age ≥18 years old with a pathologically confirmed diagnosis of cancer; (2) five or fewer cumulative extracranial metastases at the time of first SBRT course; and (3) full‐body staging or restaging imaging within 4 months of first SBRT course. Patients with brain metastases were eligible but these lesions did not contribute to the cumulative metastatic count. Patients with ≥6 cumulative sites of extracranial metastases were excluded, even when systemic therapy or other radical/ablative treatment (e,g,, metastasectomy, radiofrequency ablation) may have reduced this number to 5 or fewer.

### 2.2 Data collection and analysis

Institutional research board (IRB) approval was obtained, including data sharing agreements, prior to commencing this study. Patient demographics, disease and treatment characteristics, and disease progression assessments were retrospectively reviewed and entered into a Microsoft Access database designed for consistent use across all sites. A detailed instruction manual defining all data parameters was used to maximize consistency in data collection. Oligoprogressive events were recorded for each patient until WSP, defined as the presence of six or greater sites of untreated/active extracranial disease, or death. Additional quality assurance checks were performed by the coordinating institution to identify data inconsistencies. The data dictionary and data quality assurance schema can be found in previously published supplementary materials.^7^

For this analysis, we excluded patients from the above master database who did not have curative treatment for their primary disease, had pre‐existing brain metastases, or were not treated with local therapy to all known sites of oligometastatic disease at the time of their initial SBRT course. Any patients who were missing data for the primary outcome, primary predictor, or confounding variables were also excluded. The primary outcomes in this study were distant progression‐free survival (DPFS), defined as freedom from the development of any recurrence or progression outside the initial sites of oligometastases; widespread progression (WSP), defined as the cumulative incidence of progression with >5 sites of new disease; and overall survival (OS), defined as freedom from death from any cause. The start date of SBRT was used as the reference date for the primary outcome variables. The primary predictor in this study was the total planning target volume (PTV), defined as the sum of the PTVs of all lesions treated at the time of initial OM presentation to SBRT. The potential confounders adjusted in the multivariable regression models were primary histology, pre‐SBRT use of systemic therapy, non‐osseous metastasis versus osseous‐only, and the number of initial oligometastases, as all of these factors have been demonstrated to be associated with oncologic prognosis. For instance, osseous‐only metastatic disease appears to confer a more favorable prognosis in breast and prostate cancers.^8^, ^9^

The effect of total PTV on DPFS and OS was investigated by multivariable Cox regression, and the effect of total PTV on WSP was investigated by multivariable competing risks regression, using death as a competing risk and adjusting for the potential confounders. Total PTV was initially modeled using restricted cubic splines to investigate nonlinear effects, and significant nonlinear effects were accounted for by log transformation with stabilization of the variances after transformation. The proportional hazards assumption was assessed visually by Schoenfeld residual plots and statistically by a Schoenfeld residual test on the total PTV variable. Nonproportional hazards were accounted for by modeling total PTV with time‐dependent coefficients using categorical time intervals. A sensitivity analysis was carried out using similar methods with total gross tumor volume (GTV) instead of total PTV. Target planning expansion margins were not required to be standardized within or among participating institutions, but typical expansion margins for the participating institutions were previously reported.^10^ Briefly, the clinical target volume (CTV) expansion margin was generally 3–5 mm except for spinal lesions, which followed anatomic margins.^11^ For lung and liver lesions, 4D‐CT scans were utilized to account for internal tumor motion in defining an internal target volume (ITV). PTV expansions ranged from 3 to 5 mm except for spine metastases, which were typically limited to 1–2 mm.

Analyses were performed in R (v4.0.2 x64). A *p* value threshold of 0.05 was used for statistical significance.

## 3 RESULTS

In total, 961 patients were included in this analysis, with a median follow‐up of 24.4 months (IQR: 13.8–37.5). A summary of patient and lesion characteristics is shown in Table 1. The most common primary disease sites were NSCLC (25.9%), colorectal cancer (22.0%), prostate (13.3%), breast (7.9%), and RCC (6.2%). Most patients (72.9%) had metachronous oligometastatic disease, defined as diagnosis of metastatic disease >3 months from initial primary diagnosis. The vast majority of patients (91.6%) had three or fewer lesions at OM presentation to SBRT, and 28.3% of patients had osseous‐only initial OM disease. Approximately one third of patients (34%) had systemic therapy prior to SBRT. The mean total PTV was 66.2cc, while the median was 40cc (IQR: 19.7–85.0).

**TABLE 1:** Summary of patient and lesion characteristics

Patient and lesion characteristics	
Primary site	*n* = 961
NSCLC	249 (25.9%)
Colorectal	211 (22.0%)
Prostate	128 (13.3%)
Breast	76 (7.9%)
Renal cell carcinoma	60 (6.2%)
Sarcoma	35 (3.6%)
Melanoma	31 (3.2%)
Pancreas	25 (2.6%)
Oropharynx	20 (2.1%)
Hepatocellular	11 (1.1%)
Bladder	10 (1.0%)
Endometrium	10 (1.0%)
Larynx	9 (0.9%)
Thyroid	9 (0.9%)
Nasopharynx	6 (0.6%)
Cholangiocarcinoma/biliary	6 (0.6%)
Esophagus	6 (0.6%)
Anal	5 (0.5%)
SCLC	5 (0.5%)
Cervix	4 (0.4%)
Hypopharynx	4 (0.4%)
Gastric	3 (0.3%)
Other	38 (4.0%)
Number of metastases at oligometastatic presentation to SBRT	
1	571 (59.4%)
2	217 (22.6%)
3	92 (9.6%)
4	53 (5.5%)
5	28 (2.9%)
Timing of oligometastatic presentation	
Synchronous (≤3 months)	260 (27.1%)
Metachronous (≤24 months)	304 (31.6%)
Metachronous (>24 months)	397 (41.3%)
Pre‐SBRT systemic therapy	
Yes	327 (34.0%)
No	632 (65.8%)
Unknown	2 (0.2%)
Initial metastatic site	
Osseous‐only	272 (28.3%)
Any non‐osseous site	689 (71.7%)
All initial oligometastases treated with only SBRT	
Yes	756 (78.7%)
No	205 (21.3%)
Total GTV mean, cc (SD)	21.8 (36.0)
Total GTV median, cc (IQR)	8.2 (3.0–27.6)
Total PTV mean, cc (SD)	66.2 (74.7)
Total PTV median, cc (IQR)	40.0 (19.7–85.0)

Abbreviations: GTV, gross tumor volume; IQR, interquartile range; NSCLC, non‐small cell lung cancer; PTV, planning target volume; SBRT, stereotactic body radiation therapy; SCLC, small cell lung cancer; SD, standard deviation.

**FIGURE 1:**
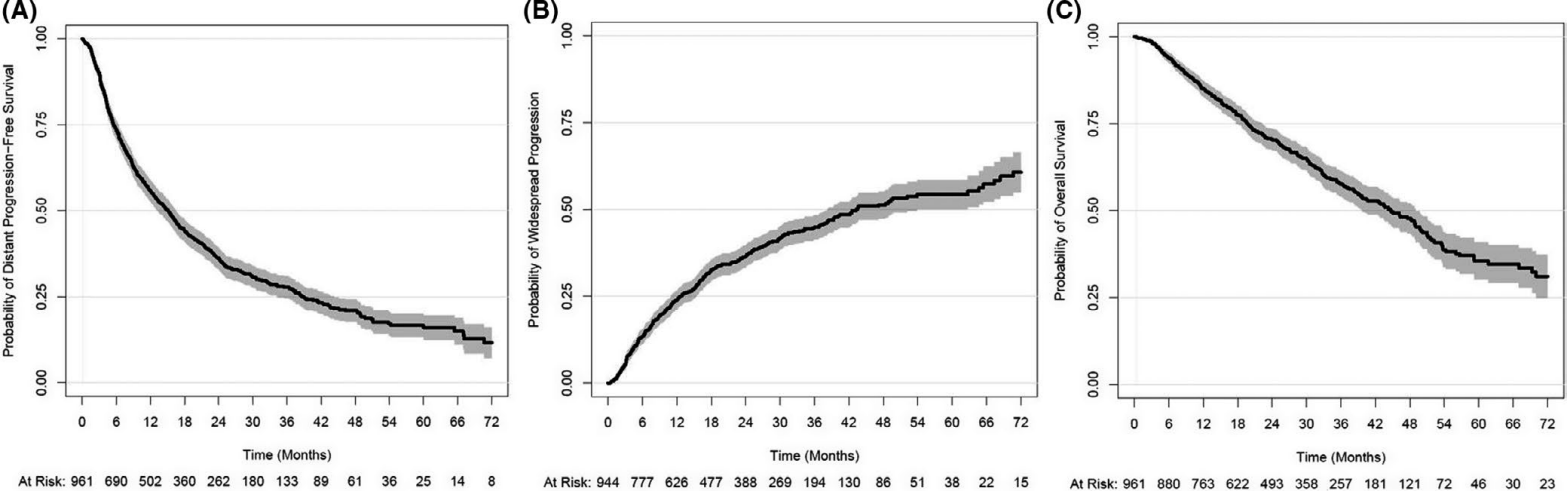
(A) Kaplan–Meier plot for distant progression‐free survival, (B) cumulative incidence plot of widespread progression, and (C) Kaplan–Meier plot for overall survival

The median DPFS was 15.1 months (95% CI: 13.3–16.6), while the median time to WSP was 43.5 months (95% CI: 38.2–53.9) and the median OS was 44.7 months (95% CI: 39.7–49.0). The representative cumulative incidence and the Kaplan–Meier plots for these three oncologic outcomes are displayed in Figure 1. Of the patients in the overall cohort who progressed during the study period, 31% presented with WSP at the time of first progression after SBRT, as detailed in a prior report summarizing outcomes.^7^

**FIGURE 2:**
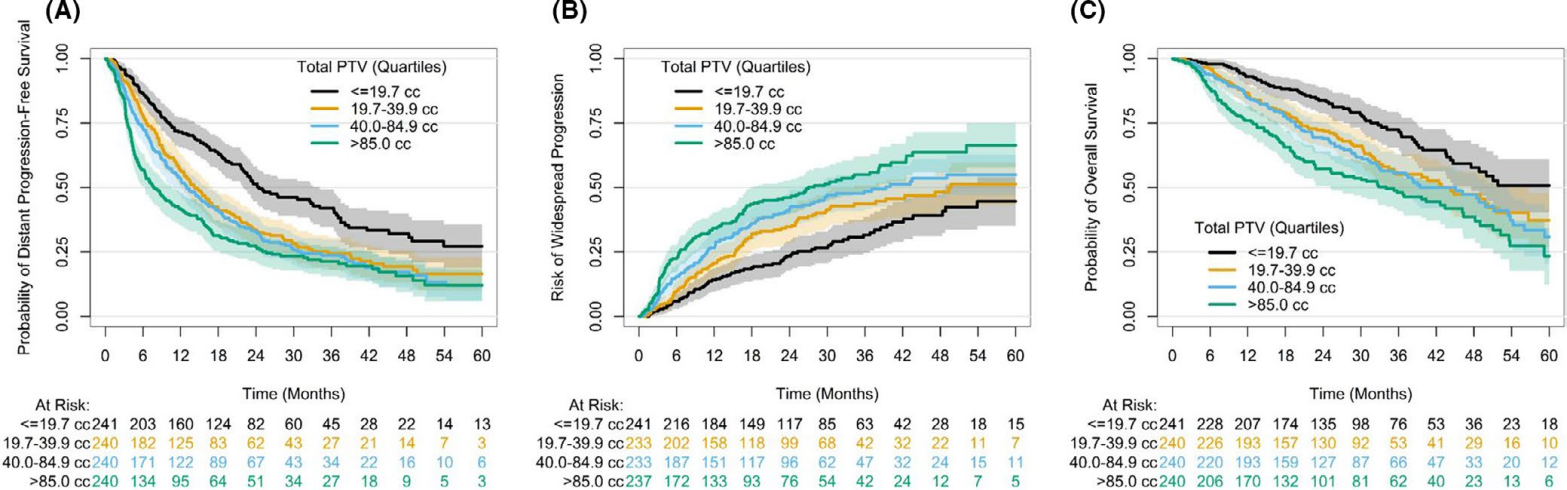
Univariable relationship of total planning target volume (PTV) per quartile with (A) distant progression‐free survival, (B) widespread progression, and (C) overall survival

After controlling for the pre‐specified confounders of histology, number of oligometastases, use of systemic therapy pre‐SBRT, and presence of osseous‐only disease, there was a significant relationship between total PTV at initial OM presentation to SBRT and each of these oncologic outcomes after MDRT, which is summarized in Table 2. The Schoenfeld residual test showed significant nonproportional hazards. The effect of PTV on DPFS was significant in the 18 months after SBRT, where each twofold increase in total PTV increased the risk of distant progression by 40.6% (95% CI: 28.1–54.3%, *p*<0.0001) in the first 6 months, by 11.6% with borderline significance during months 6–12 (95% CI: −0.7–25.4%, *p *= 0.066), and by 18.2% in months 12–18 (95% CI: 2.2–36.7%, *p *= 0.024). Meanwhile, the relationship of total PTV and risk of WSP was only significant in the first 6 months after SBRT, where each twofold increase in total PTV increased the risk of WSP by 45.4% (95% CI: 27.7–65.7%, *p *< 0.0001). Lastly, the relationship between total PTV and OS maintained significance during the first 2 years after SBRT, where each twofold increase in total PTV increased the risk of death by 60.7% in the first 6 months (95% CI: 32.7–94.7%, *p *< 0.0001), and by approximately 34% within each 6‐month period over the next 18 months (*p *< 0.01). The relationships between total PTV and each oncologic outcome are visually presented in Figure 2, as stratified by the total PTV quartiles in our cohort.

**TABLE 2:** Results of multivariable regression models for distant progression, widespread progression, and overall survival

	Distant progression			Widespread progression			Overall survival		
Multivariable	HR	95% CI	*p* value	HR	95% CI	*p* value	HR	95% CI	*p* value
Total PTV (per twofold increase) based on time from SBRT									
0–6 months	1.406	1.281–1.543	<0.0001	1.454	1.277–1.657	<0.0001	1.607	1.327–1.947	<0.0001
6–12 months	1.116	0.993–1.254	0.066	1.156	0.993–1.345	0.061	1.347	1.146–1.582	<0.001
12–18 months	1.182	1.022–1.367	0.024	1.106	0.939–1.304	0.229	1.342	1.121–1.607	<0.01
18–24 months	0.933	0.772–1.128	0.472	1.074	0.832–1.386	0.584	1.349	1.109–1.641	<0.01
24–36 months	1.089	0.893–1.329	0.399	1.062	0.880–1.282	0.530	1.109	0.933–1.318	0.241
36–48 months	0.975	0.747–1.272	0.851	1.098	0.821–1.470	0.528	1.033	0.809–1.318	0.794
>48 months	0.978	0.702–1.362	0.893	0.655	0.451–0.951	0.026	1.117	0.869–1.436	0.388
Primary site									
Breast (Reference)	1.000			1.000			1.000		
Colorectal	1.280	0.895–1.830	0.177	1.078	0.690–1.683	0.742	1.368	0.827–2.262	0.222
NSCLC	1.539	1.087–2.178	0.015	1.114	0.715–1.734	0.634	2.569	1.596–4.135	<0.001
Renal cell carcinoma	1.278	0.840–1.944	0.252	0.683	0.390–1.193	0.180	0.962	0.516–1.794	0.904
Prostate	0.591	0.402–0.869	0.007	0.473	0.297–0.754	0.002	0.274	0.128–0.588	<0.001
Other	2.365	1.678–3.333	<0.0001	1.516	0.994–2.313	0.054	2.842	1.774–4.553	<0.0001
Systemic therapy prior to SBRT (yes vs. no)	1.187	0.993–1.420	0.060	1.395	1.102–1.767	0.006	1.081	0.854–1.370	0.517
Any non‐osseous metastasis vs. osseous‐only metastasis	0.832	0.665–1.041	0.108	0.749	0.555–1.009	0.057	1.063	0.795–1.419	0.681
Number of metastases									
1 (Reference)	1.000			1.000			1.000		
2	1.143	0.945–1.383	0.168	1.245	0.979–1.582	0.074	1.201	0.97–1.582	0.138
3	1.472	1.140–1.899	0.003	1.158	0.823–1.627	0.400	0.902	0.626–1.299	0.579
4	1.196	0.854–1.675	0.297	1.527	1.030–2.264	0.035	1.158	0.734–1.827	0.528
5	1.082	0.681–1.720	0.739	1.496	0.890–2.514	0.129	0.842	0.441–1.608	0.603

Abbreviations: NSCLC, non‐small cell lung cancer; PTV, planning target volume; SBRT, stereotactic body radiation therapy.

**FIGURE 3:**
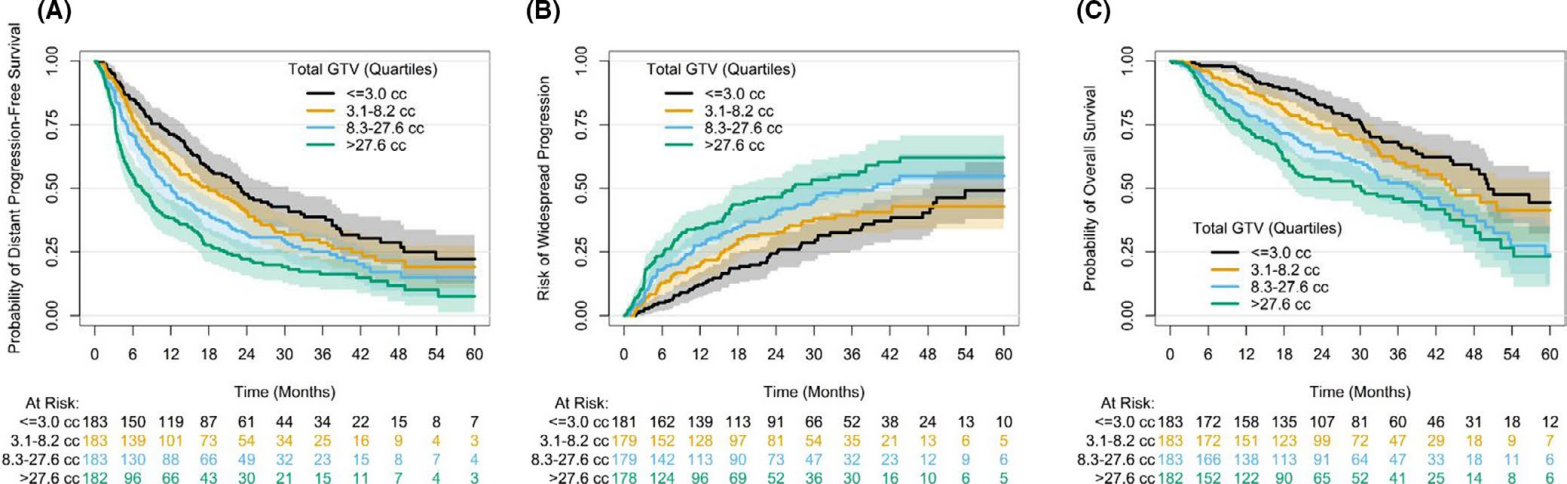
Univariable relationship of gross target volume (GTV) per quartile with (A) distant progression‐free survival, (B) widespread progression, and (C) overall survival

Complete gross tumor volume (GTV) data were available for 76.1% of patients and were unavailable in some metastatic sites where target delineation may favor CTV or ITV over GTV alone. For instance, 54.1% of spine lesions and 30.3% of lung lesions were missing GTV values. An exploratory multivariable analysis of the available total GTV data confirmed similar significant associations between tumor volume and DPFS, WSP, and OS after adjusting for the same pre‐specified confounders. Briefly, each twofold increase in total GTV increased the risk of distant progression by 26.6%, WSP by 26.5%, and death by 44.9% in the first 6 months (*p *< 0.0001). The effect of increasing GTV on distant progression and OS remained significant during the first 12 and 18 months after SBRT, respectively. The association of total GTV and each oncologic outcome is visually demonstrated in Figure 3, which shows a pattern of separation of curves when stratifying by total GTV quartiles that is similar to what is seen in the PTV data.

## 4 DISCUSSION

We report a novel and impactful relationship of total metastatic volume with cancer outcomes in oligometastatic patients, which should be used to refine the current definition of oligometastatic disease. As the oligometastatic state becomes increasingly recognized, the central question is that of optimal patient selection to leverage this unique therapeutic opportunity. The ultimate goal of classifying oligometastatic disease is to identify patients who (1) develop a limited number of metastatic deposits and (2) whose disease does not progress to widespread progression. This is in order to select for those who may benefit most from maximally aggressive local therapy as opposed to systemic therapy alone, which can improve survival but is rarely curative for solid tumors.^12^

Many different metrics have been proposed to identify the oligometastatic state, with the most widely adopted being number of metastatic lesions, typically capped at five lesions or fewer. A numerical definition is supported by early data in metastasectomy. For instance, a study of over 5,000 patients with epithelial cancers and sarcomas in the International Registry of Lung Metastases demonstrated that patients with solitary lung lesions had a 5‐year OS of 43%, in contrast to 27% for those with ≥4 lesions.^13^ Nonetheless, the "3"or "5" number that the oncologic community has widely embraced, at best, is informed by clinical acumen and retrospective series like the aforementioned, and at worst, represents an arbitrary number with limited biological basis. While SBRT in an OM treatment paradigm has been increasingly utilized, identifying the ideal patient candidates remains quite subjective. Therefore, the purpose of this study was to evaluate if the total metastatic volume at initial OM presentation to SBRT could add to a quantitative definition for guiding oligometastases management. In our large, multi‐institutional series, we found that volumetric metastatic burden, quantified as the summed volume of all SBRT‐targeted PTVs, was independently prognostic for distant progression‐free survival, widespread progression, and overall survival in a time‐dependent fashion. Importantly, this remained true even after adjusting for key confounders like histology, number of oligometastases, osseous‐only versus any non‐osseous metastasis, and the use of any systemic therapy prior to SBRT.

It is well‐established that size of the primary tumor at diagnosis influences the risk of metastasis across many tumor types and therefore is commonly incorporated into the TNM staging system. An NCDB (National Cancer Database) analysis of over 300,000 colon cancer patients found an association between tumor size and nodal metastasis rate; 79% of patients with tumors ≤2 cm were node‐negative compared to 48% of patients with tumors >6 cm.^14^ Similarly, a SEER (Surveillance, Epidemiology, and End Results) database analysis of over 800,000 invasive breast cancer patients found that the prevalence of distant metastases at diagnosis ranged from 0.5% for primary tumors ≤1 cm in size and up to 26.3% for tumors measuring between 9 and 10 cm. However, the authors observed a plateau in “metastatic potential” beyond a certain size, arguing that the primary tumor is unlikely to be the sole metastatic source if patients with 7 cm primary tumors demonstrate the same lymph node metastasis rate as patients with 15 cm tumors (~71%), as was their observation.^15^

This speaks to the conflict between the two leading traditional models of metastasis. The first is the linear progression model, in which metastasis is unidirectional. In this model, cancer cells within the primary tumor acquire mutational and growth capacities over time to gain distant metastatic potential. The parallel progression model, on the other hand, describes metastasis as an early event marked by potential periods of dormancy. Metastases and primary tumors are considered thereafter to evolve independently of one another.^16^ A more recent theory of metastatic behavior has been termed “tumor self‐seeding,” acknowledging that metastatic cells evolve in the primary tumor but also in distant sites, possessing even the ability to return to the primary tumor and “seeding” it with its own biologically aggressive progeny.^17^, ^18^ Certainly, the ability to differentiate among these metastasis models is far beyond the scope of this project, but if we consider metastasis to be a much less unidirectional and linear process than traditionally thought, it is no surprise that the size of metastatic lesions correlates with important outcomes in oligometastatic patients. The most intuitive explanation is that if metastatic lesions themselves are able to meaningfully seed new metastases (and potentially even selecting for more aggressive phenotype), then larger metastases may have higher metastatic potential similar to the pattern seen with larger primaries. Another theory stems from the fact that our ability to detect metastatic disease is highly limited by our existing radiographic techniques, and that with increasing size (and number) of oligometastases, the likelihood of occult disseminated disease increases.^19^

Our findings for extracranial metastases mirror similar patterns seen in intracranial metastatic disease, where the cumulative intracranial tumor volume has been shown to be an independent prognostic factor for overall survival following stereotactic radiosurgery, where interestingly, number of brain metastases was not found to be predictive.^20^ Hirshman *et al* showed that cumulative intracranial tumor volume was found to have superior prognostic value in predicting 1‐year survival when replacing the largest intracranial tumor volume in the Score Index for Radiosurgery model.^21^ However, data in *extracranial* metastatic disease speaking to the impact of size/volume of metastatic lesions on patient outcomes are very limited. One report did show in differentiated thyroid carcinoma with metastases isolated to the lung that the largest diameter of any existing metastatic lesion was the most important prognostic factor for progression‐free and cancer‐specific survival, even when accounting for other important risk factors like primary tumor size, extra‐thyroidal invasion, regional/cervical lymph node metastasis, and radioactive iodine (RAI) activity.^22^ Our findings support total volume of extracranial metastatic lesions as a primary factor in defining the oligometastatic state, and helps to fill a void in the data for guiding the increasingly important oligometastatic treatment paradigm.

This study is subject to inherent biases of patient and treatment selection as a retrospective analysis. While histology was included in the multivariable analysis, we recognize that our findings may not be as generalizable to the more underrepresented histologies in our cohort. However, this study does leverage the strengths of a multi‐institutional study in building a relatively large and diverse patient cohort, for which we attempted to optimize consistent reporting among institutions through use of a comprehensive data dictionary and several layers of data quality checks, as previously detailed.^7^ Nonetheless, there were institutional differences in target delineation procedures, such as the use of GTV versus ITV or CTV in treatment planning, leading to gaps in data availability for GTV, arguably the most intuitive representation of tumor volume. This led us to use the more uniformly available PTV metric as our tumor volume proxy. We acknowledge that due to institutional differences in PTV expansion margins, this introduces inconsistencies in the representation of true tumor volume, with variations highly dependent on the metastatic site (i.e., particularly for spine SBRT where CTV is more commonly used, or for lung SBRT where ITV is often utilized). However, we are reassured by our exploratory GTV analysis demonstrating very similar and highly statistically significant relationships, and thereby confirming the findings from the PTV analysis. Another limitation is that a minority of our patients (21.3%) had prior non‐SBRT management of OM (e.g., metastasectomy) and the volume of those lesions was not captured in our analysis. However, this reflects the reality of the variations in initial presentation of suspected OM patients to multidisciplinary management, particularly when the metastatic state first needs to be pathologically confirmed.

Our findings specific to the initial OM presentation to metastasis‐directed radiotherapy are important in guiding this increasingly popular paradigm and may help to identify patients who would most benefit from systemic therapy in addition to local therapy. A larger volume of metastatic disease may, for instance, be a promising surrogate marker for more aggressive tumor biology or higher risk of occult disseminated micrometastatic disease, even when the oligometastatic definition appears to be satisfied by the number of gross macroscopic lesions. The prognostic value appears to be restricted to the first several months to years after initial SBRT, suggesting that other clinical factors drive prognosis as patients progress further into their disease course. However, the significant association with oncologic outcomes in the immediate period after SBRT, particularly striking in the first 6 months, suggests that this could become a key indicator of early disease behavior and differentiate the “oligometastatic” patients at highest risk of early widespread progression or death, for whom systemic and multimodality therapy should be recommended. Total metastatic volume has the potential to serve as a powerful multidisciplinary decision‐making tool but first needs to be validated in disease histology‐specific external datasets and prospective studies.

In conclusion, we demonstrate a novel and significant time‐dependent relationship between total metastatic volume and distant progression‐free survival, widespread progression, and overall survival in patients with extracranial oligometastatic disease. Total metastatic volume should be incorporated as an important independent clinical factor for evaluating oligometastatic disease as we move into an era with increasingly aggressive utilization of metastasis‐directed radiotherapy for stage IV cancer patients. We show that total volume of metastatic lesions has prognostic merit beyond number of metastases in identifying OM patients, a concept that warrants further study and incorporation into prospective clinical trials.

## CONFLICT OF INTEREST

Y.C. and H.C. have no conflict of interest to declare. All other authors reported receiving travel support from Elekta AB through the Consortium for Oligometastases Research (CORE). A.S. has performed educational seminars with Medtronic, Elekta AB, Accuray Inc., and Varian medical systems, has received research grants and travel expenses from Elekta AB, and is part of the Elekta MR‐Linac research consortium. T.B. reported advisory board positions with Galera Therapeutics and AstraZeneca. R.D. has received research grants from Elekta AB. M.F. has received honoraria, research grants, and travel expenses from Elekta AB. A.V.L. has received honoraria from Varian medical systems, AstraZeneca, and RefleXion. I.P. has received research grants from Elekta AB and has held advisory board positions with Sanofi Aventis and AstraZeneca. K.J.R. has received research support, travel expenses, and honorarium for educational seminars from Accuray; participates in a data safety monitoring committee for BioMimetix; and received travel expenses from Brainlab and Elekta AB.

## Data Availability

The data that support the findings of this study are available upon request from the corresponding author. The data are not publicly available due to privacy or ethical restrictions.
